# Natural Occurrence, Bioactivity and Biosynthesis of Elaiophylin Analogues

**DOI:** 10.3390/molecules24213840

**Published:** 2019-10-25

**Authors:** Min Gui, Meng-xue Zhang, Wen-hui Wu, Peng Sun

**Affiliations:** 1 State Key Laboratory of Dairy Biotechnology, Technology Center and Dairy Research Institute of Bright Dairy & Food Co. Ltd., 1518 West Jiangchang Road, Shanghai 200436, China; glemine@hotmail.com; 2 School of Pharmacy, Second Military Medical University, 325 Guohe Road, Shanghai 200433, China; yzmx1216@163.com; 3 College of Food Science and Technology, Shanghai Ocean University, 999 Huchenghuan Road, Shanghai 201306, China

**Keywords:** elaiophylin, efomycin, macrodiolide, C_2_-symmetry, antimicrobial, anticancer, immunosuppressive, biosynthesis

## Abstract

Elaiophylins belong to a special family of 16-membered macrodiolides with C_2_-symmetry. They have exhibited remarkable biological activities, such as antimicrobial, anthelmintic, anticancer, immunosuppressive, anti-inflammatory, antiviral, and α-glucosidase inhibitory activities. A member of elaiophylins, efomycin M, is as a specific small molecule inhibitor of selectin in preclinical trial for the treatment of psoriasis, ischemia-reperfusion, and allergy. The biosynthetic investigation of elaiophylins has uncovered a unique thioesterase, which is responsible for the formation of C_2_-symmetric diolide. We herein summarize the natural occurrence, bioactivity, and biosynthesis of elaiophylins covering the literatures from 1959 to 2019. Hopefully, this review will inspire further research interests of these compounds and encourage the discovery of new analogues by metabolic engineering or genome mining.

## 1. Introduction

The macrolides are a group of antibiotics whose activity stems from the presence of a large macrocyclic lactone ring to which one or more deoxy sugars may be attached. Normally, macrolides are assembled by the polyketide synthases (PKSs), which are large multifunctional enzymes that are composed of multiple discrete domains, each being responsible for catalyzing a single reaction of the polyketide biosynthetic process [1]. Macrodiolides, often classified into a small group of macrolides, represent a heterogenous group of microbial metabolites whose common feature is the presence of a cyclic diester moiety in their structure [2]. To date, only several families of macrodiolide natural products have been discovered, including elaiophylins [3], conglobatins [4], samroiyotmycins [5], bispolides [6], vermiculin [7], and pyrenophorin [8], from actinomycetes or fungi. Among them, elaiophylins are 16-membered glycosylated macrodiolides featuring with unusual C_2_-symmetry and hemiketal moiety. Elaiophylins have displayed a wide range of biological properties, such as antimicrobial, anthelmintic, anticancer, immunosuppressive, anti-inflammatory, antiviral, and α-glucosidase inhibitory activity. The anticancer and immunosuppressive activities have attracted extensive interests for further mechanism investigation. Efomycin M, as a new type of selective inhibitor of selectin functions, is currently in preclinical trial for the treatment of psoriasis, ischemia-reperfusion, and allergy. In this review, we summarize the natural occurrence, biological activity, the structure-activity relationship, and biosynthesis of elaiophylin family with structures that were derived from different *Streptomyces* species covering literatures from 1959 to 2019. The chemical synthesis of elaiophylins is not included.

## 2. Natural Occurrence

The first member, elaiophylin (**1**, also known as azalomycin B, salbomycin, efomycin E, or gopalamycin), was isolated from a culture of *Streptomyces melanosporus* in 1959 [3,9,10,11] and obtained as azalomycin B in 1960 [12]. The structure was assigned by chemical degradation, analyses of NMR and MS spectroscopic data [9,13,14]. Its absolute configuration was determined by a X-ray crystallographic analyses [15]. Later on, efomycin E derived from *Streptomyces* sp. BS 1261 and gopalamycin isolated from *Streptomyces hygroscopicus* MSU-625 and MSU-616 were found to be identical to elaiophylin [16,17]. Gopalamycin was originally assumed not to be elaiophylin, because it showed significant antifungal activity that elaiophylin did not have. However, their structures are the same according to the X-ray crystal analysis [17]. Subsequently, elaiophylin was found to be produced from various *Streptomyces* species [18]. Sporulation conditions of *Streptomyces melanosporofaciens* were optimized to maximize the concentration of elaiophylin at 1.2 g/kg fermentation medium and a described procedure resulted in the preparation of elaiophylin of pharmaceutical grade [19].

**Figure 1:**
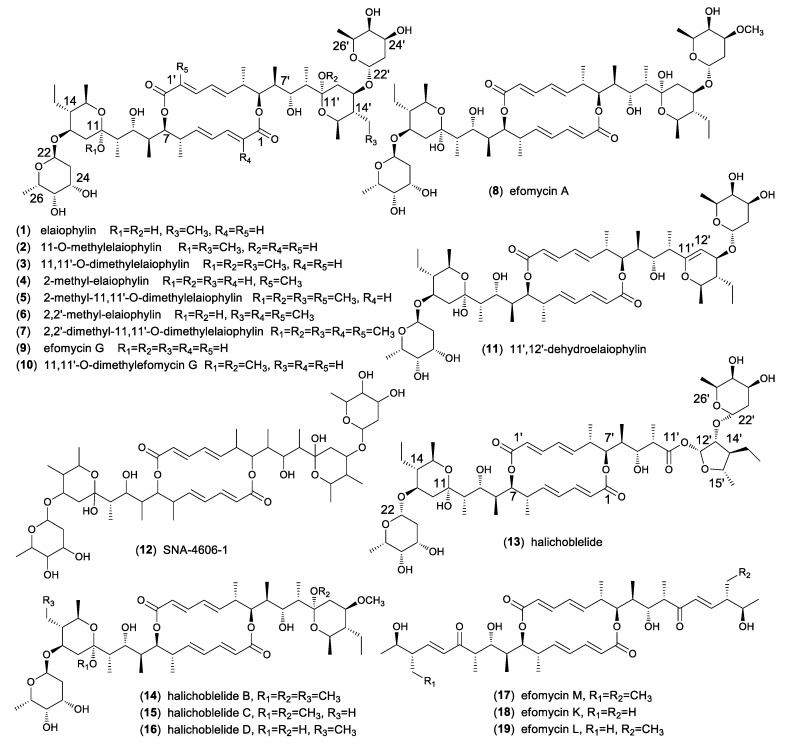
Structures of naturally occurring elaiophylins.

Along with the discovery of elaiophylin, eighteen analogues were obtained from different *Streptomyces* spp as shown in Figure 1. 11-O-methylelaiophylin (**2**) and 11,11’-O-dimethylelaiophylin (**3**) were isolated from the mycelium cake of *Streptomyces* strains HKI-0113 and HKI-0114. The structures were determined by MS and NMR spectrometric investigations [20]. From cultures of the Indonesian soil *Streptomyces* sp. ICBB 9297 four new elaiophylin macrolides, 2-methyl-elaiophylin (**4**), 2-methyl-11,11’-O-dimethylelaiophylin (**5**), 2,2’-dimethyl-elaiophylin (**6**), and 2,2’-dimethyl-11,11’-O-dimethylelaiophylin (**7**), along with **1**–**3** were isolated. Their structures contain an additional methyl groups at C-2 or C-2’, which are different from other elaiophylin analogues [21]. The Bayer company researchers reported the purification of efomycin(e)s A-F from the culture of a soil-derived *Streptomyces* sp. BS1261 and their mixtures showed potential as performance promoters in farm animals and exhibited antibacterial and antiviral activities [22]. The chemical and physical properties of efomycins A-F and their NMR data were reported. Among them, efomycin E were identical to elaiophylin and efomycin A (**8**) differed from elaiophylin by the methoxy substitution at C-24′. However, the structures of efomycin B, C, D, and F remained unassigned. From the same strain, efomycin G (**9**) was later isolated which possesses a methyl group at C-14’ in contrast to an ethyl group in elaiophylin [23] 11,11’-O-dimethylefomycin G (**10**) and 11′,12′-dehydroelaiophylin (**11**) were purified from culture extracts of a marine sediment derived strain *Streptomyces* sp. 7-145 by a PCR-based genetic screening experiment targeting the dTDP-glucose-4,6-dehydratase gene [24]. SNA-4606-1 (**12**) was isolated as an enzyme inhibitor from the cultured broth of *Streptomyces* sp. SNA-4606 along with **1** and **9**. The structure was determined by NMR spectroscopic data, which showed the presence of methyl groups at both C-14 and C-14’. However, its relative configuration was not determined [25]. Halichoblelide (**13**) had been isolated from *Streptomyces hygroscopicus* originally separated from the marine fish *Halichoeres bleekeri*. Its structure and absolute configuration was elucidated by application of NMR spectroscopic analyses, the modified Mosher’s method, and CD spectra of the other products [26]. Two analogs, halichoblelides B (**14**) and C (**15**), were isolated from the same strain with one of the 6-deoxyfucose units being replaced by a methoxy group. Their absolute configurations were elucidated on the basis of spectroscopic analyses using one-dimensional (1D) and two-dimensional (2D) NMR techniques and chemical transformations [27]. Halichoblelide D (**16**) was separated and identified from the culture broth of mangrove strain *Streptomyces* sp. 219807. Its absolute configuration was determined by comparing the CD spectrum with those of the reported analogues [28]. Efomycin M (**17**) was originally prepared by base-catalyzed β-elimination of the deoxyfucose side chains of the mixture of efomycins [29]. Later, it was isolated from a rice endophytic actinomycete strain *Streptomyces* sp. BCC72023 along with efomycin G (**9**) [30]. Efomycin M differs from other elaiophylins by the absence of glycosylated dihydroxypyrane moiety and the presence of an unsaturated enone. As part of the explorations for antifungal activity of *Streptomyces* sp. M56 against both the termite mutualistic cultivar (*Termitomyces* spp.) and competitors/antagonists of this cultivar (*Pseudoxylaria* spp.), two new elaiophylin analogues, efomycins K (**18**) and L (**19**), and five known derivatives (**1**–**3**, **9**) were isolated. Similar to efomycin M, **18** and **19** carried an unsaturated enone moiety and the structures were determined by analyzing NMR and HR-ESIMS data and by comparative CD spectroscopy [31].

The elaiophylin family shows high structural similarity in the diolide backbone, the hemiketal and 6-deoxyfucose moieties. Most structural variations of elaiophylin exist in different substitution patterns, such as proton or methyl groups at C-2 and/or C-2′, hydroxy, or methoxy groups at C-11 and/or C-11′, as well as methyl or ethyl groups at C-14 and/or C-14′. In contrast to elaiophylin (**1**) halichoblelide B-D (**14**–**16**) have commonness where one 6-deoxyfucose unit at C-13’ is replaced by a methoxy group. Efomycins K-M (**17**–**19**) are unique by the absence of glycosylated hemiketal moiety and the presence of unsaturated enone. The elaiophylin analogues that have methoxy substitutions at C-11 and/or C-11′ might be artifacts when MeOH was used during isolation and purification processes [32]. For example, when exposed to MeOH, the natural product elaiophylin (**1**) undergoes stereospecific acetal alkylation to 11-O-methylelaiophylin (**2**) and 11,11’-O-dimethylelaiophylin (**3**), while the co-metabolite efomycin G (**9**) undergoes transformation to 11,11’-O-dimethylefomycin G (**10**) [21].

## 3. Biological Activities

Elaiophylin and its derivatives have exhibited a myriad of activities, including antimicrobial, anthelmintic, anticancer, immunosuppressive, anti-inflammatory, antiviral, and α-glucosidase inhibitory activities. The mechanisms of elaiophylins on anticancer and immunosuppressive activities have been extensively explored. The relationship between the structure and biological activity of elaiophylin derivatives has also been studied, as outlined below.

### 3.1. Antimicrobial and Anthelmintic Activity

Elaiophylins exhibited antimicrobial activities against Gram-positive bacteria, but it was not active against Gram-negative bacteria, yeast, or fungi. Lee et al. reported an antimicrobial spectrum for elaiophylin with a concentration up to 100 μg/mL [33]. Although elaiophylin (**1**) has no activity against *Candida albicans*, it was able to markedly enhance rapamycin’s antifungal activity [34]. The mechanism of antibacterial activity of elaiophylin is not clear. Probably, it is related to the ability to form stable, long-lasting cation selective ion channels in microbial bilayer membranes [35].

The antimicrobial activities of 11-O-methylelaiophylin (**2**) and 11,11′-O-dimethylelaiophylin (**3**) were evaluated in an agar plate diffusion assay. They both showed antimicrobial activity against Gram-positive bacteria *Bacillus subtilis*, *Staphylococcus aureus*, *Mycobacterium* sp. SG, and *Enterococcus faecium* at the concentration of 50 μg/mL, but were not active against *C. albicans* [20]. In another biological test, **3** displayed lower antimicrobial activity than **2**, which suggested the importance of free hemiketal hydroxyl in the antimicrobial activity [36]. Sheng et al. reported that compounds **4**–**7** displayed comparable antibacterial activity to **1** against *S. aureus*, with minimum inhibitory concentration (MIC) values in the range of 0.78–3.13 μg/mL, but did not inhibit the growth of *B. subtilis*, *Pseudomonas aeruginosa*, or *Escherichia coli,* with a concentration up to 100 μg/mL. Compounds **4** and **5**, which have only one pendant methyl group at C-2, showed good activities against *M. smegmatis* with an MIC value of 6.25 μg/mL, whereas compound **7**, which contains two methyl groups, and compounds **1**–**3**, which do not have the C-2 and/or C-2′ methyl groups, showed no growth inhibition [21]. Efomycin G (**9**) exhibited antibacterial activity against *Plasmodium falciparum*, *M. tuberculosis* H37Ra, *B. cereus*, with MIC values of 2.37, 12.0, and 3.13 μg/mL, respectively. Efomycin M (**17**) showed antimalarial activity against *Plasmodium falciparum* with a MIC value of 5.23 μg/mL [30].

The elaiophylin (**1**) and its analogues (**2**, **3**, **9**, **10**, and **11**) were also evaluated for antibacterial activities against drug-resistant pathogens, including methicillin-resistant *S. aureus* (MRSA) and vancomycin-resistant *enterococci* (VRE) strains (Table 1). Particularly, compounds **1**, **2**, **9**, and **11** showed potent activity with MIC values of 1–4 μg/mL against a number of MRSA and VRE strains, most of which were highly resistant (MIC > 256 μg/mL) to the controls. Furthermore, compounds **1, 2, 3, 9** inhibited methicillin resistant *S. epidermidis* (MRSE), with MIC values in the range of 2–16 μg/mL. The lack of cross-resistance between these compounds and erythromycin and azithromycin indicated that elaiophylins are probably not affected by the common macrolide resistance mechanism in Gram-positive bacteria. In comparison to **1**, the 11-methoxylated derivative (**2**) suffered from a roughly two-fold decrease in activity against most strains (MIC values 1–2 vs. 2–4 μg/mL). Furthermore, the 11,11′-dimethoxylated derivatives **3** and **10** showed dramatic 8- to 32- fold reductions as compared to **1** and **9** with OH-11 and OH-11′ (MIC values 8–64 vs. 1–2 μg/mL), respectively. These results highlight the importance of the hemiketal moiety with respect to antibacterial activity. In addition, the replacement of ethyl substituent at C-14′ by a methyl group resulted in a 2- to 4- fold decrease in potency (**1** vs. **9**; **3** vs. **10**), which indicates that the C-14/C-14′ alkyl group might also be crucial for antibacterial activity [24].

**Figure 2:**
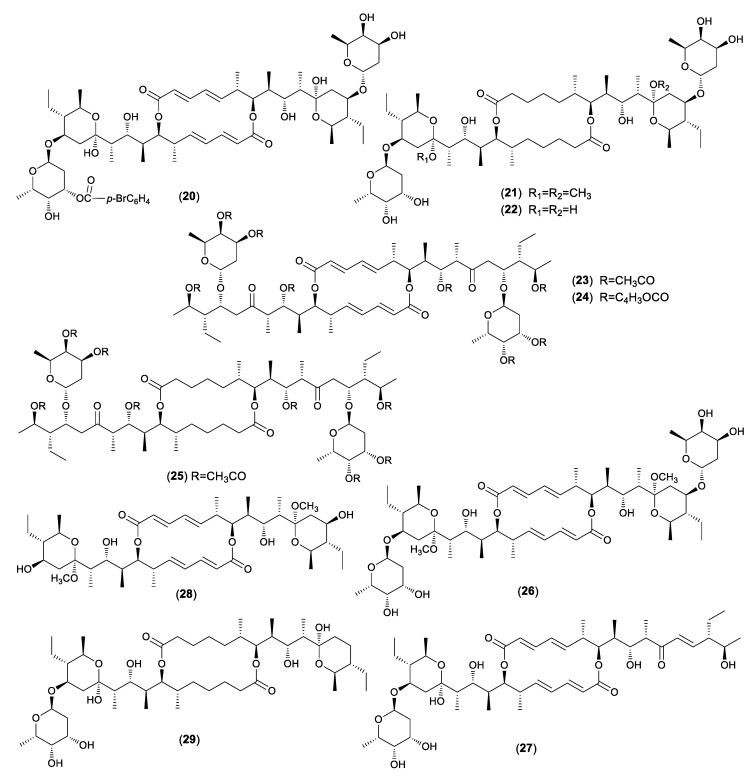
Structures of chemical derivatives of elaiophylin.

Hammann et al. described the chemical transformation of 34 acyl derivatives and six de-glycosidation products of elaiophylin (**1**) and antibacterial evaluation against Gram-positive bacteria (Figure 2) [37]. The acetalization of **1** to **2** led to a 50% reduction of antibacterial activity. All of the tetra-acyl derivatives were mainly inactive. The bromobenzoylation of **1** showed that the introduction of one acyl group (**20**) remained some activity against bacteria. However, in the higher acylated products, the activity was totally lost. The dimethyloctahydroelaiophylin (**21**) exhibited antibacterial activity, but, surprisingly, the octahydroelaiophylin (**22**) was inactive. The products with the open hemiketal ring (**23**–**25**) exhibited no activity. While the unsymmetric de-glycosidation products (**26**, **27**) had small antibacterial activity, the symmetric de-glycosidation compounds (**17**, **28**) were inactive. The remained activity of some derivatives could be explained by the fact that one part of the molecule still had an intact hemiketal moiety. Furthermore, compound **21** and the de-glycosidation products (**25**, **27**, **29**) expressed strong anthelmintic activity against the nematode *Caenorhabditis elegans* at a concentration of 100 ppm.

### 3.2. Anticancer Effect

#### 3.2.1. Cytotoxicity.

Elaiophylin (**1**) showed moderate cytotoxicities against mouse embryo fibroblast (NIH-3T3), ras transformed-NIH3T3 (F25), human gastric cancer (SNU-1), human hepatocellular carcinoma (SNU-354), viblastine sensitive human epidermoid carcinoma (KB-3-1), and resistant cervical cancer (KB-V1) cell lines, with IC_50_ values in the range of 0.39–4.40 μM. However, the compound did not show any differential effect between these cells [33]. 11-O-methylelaiophylin (**2**) and 11,11′-O-dimethylelaiophylin (**3**) displayed cytotoxicity against L929 mouse fibroblast cells, K562 human leukemia cells, and HeLa cell cultures with IC_50_ values in the range of 0.7–2.4 μg/mL. In comparison to **1**, **2** and **3** displayed somewhat lower cytotoxicity [20]. In another cytotoxic bioassay, **1** and **2** showed comparable cytotoxicities against human lung adenocarcinoma (A549), human prostatic carcinoma (PC-3), human breast adenocarcinoma (MCF-7 and MCF-7/ADR), mouse fibroblast (L929), NIH-3T3, F25, SNU-1, SNU-354, KB-3-1, and KB-V1 cell lines with adriamycin, but **3** was less active than **1** and **2** [36]. These results indicated that the OH-11 or OH-11′ of hemiketal was important for cytotoxic activity. Halichoblelide (**12**) showed potent cytotoxicity against the murine P388 lymphocytic leukemia cell line (ED_50_ = 0.63) and 39 human cancer cell lines. The tested mean value of log GI_50_ over all cell lines appeared to be −5.25 [26]. Halichoblelides B (**13**) and C (**14**) also exhibited significant cytotoxic activity against the P388 cell and, in addition, appreciable cytotoxicity against a disease-oriented panel of 39 human cancer cell lines [27]. Han et al. had evaluated the cytotoxic activities of halichoblelide D (**15**) as well as **1**–**5,** which exhibited IC_50_ values ranging from 0.19 to 2.12 μM [28]. Efomycin G (**9**) and M (**17**) were evaluated for cytotoxicity against MCF-7, KB, and human small-cell lung cancer (NCI-H187) and non-cancerous (Vero) cells, with IC_50_ values of 10.34, 23.50, 8.03, 5.37 and 5.16, 7.86, 1.56, 3.68 μg/mL [30].

#### 3.2.2. Autophagy Inhibitory Activity.

Elaiophylin (**1**) was identified as a novel autophagy inhibitor, with a significant antitumor effect as a single agent or in combination in human ovarian cancer cells. It can promote autophagosome accumulation, but block autophagic flux by attenuating lysosomal cathepsin activity, resulting in the accumulation of SQSTM1/p62 in various cell lines. Moreover, **1** can destabilize lysosomes, as indicated by CTSB/cathepsin B and CTSD/ cathepsin D release from lysosomes into the cytoplasm. Elaiophylin decreases cell viability, especially when with cisplatin or under hypoxic conditions. Furthermore, the administration of a lower dose (2 mg/kg) of **1** achieved a significant antitumor effect without toxicity in an orthotopic ovarian cancer model with metastasis. However, high doses (8 mg/kg) of **1** lead to dysfunction of Paneth cells, which resembled the intestinal phenotype of ATG16L1-deficient mice [38].

#### 3.2.3. Inducing Apoptosis and Proliferation Activity.

Elaiophylin (**1**) has been assessed on multiple myeloma (MM) cells with mutant TP53. The results indicated that 1 exerted anti-myeloma activity by inducing apoptosis and proliferation arrest. Elaiophylin blocked autophagy flux in MM cells and subsequently induced the persistent activation of endoplasmic reticulum stress. In vivo studies revealed that **1** could suppress MM cell growth without obvious side effects in zebra fish embryo and mouse xenograft models. The exposure of human MM cells with mutant TP53 to **1** blocked autophagy flux and induced cell death, which partially involved ER stress-associated apoptosis. Therefore, it is a promising therapeutic strategy that **1** can targeted disrupt the cellular protein handling system for overcoming incurable MM, even with TP53 mutations [39].

#### 3.2.4. Antiangiogenic Activity.

Elaiophylin (**1**) was demonstrated to have potent antiangiogenic activity from both in vitro and in vivo angiogenesis assays. Elaiophylin dramatically suppressed in vitro angiogenic characteristics, such as proliferation, migration, adhesion, invasion, and tube formation of human umbilical vein endothelial cells (HUVECs) stimulated by vascular endothelial growth factor (VEGF) at non-toxic concentrations. In addition, **1** immensely inhibited in vivo angiogenesis of the chorioallantoic membrane from growing chick embryos without cytotoxicity. The activation of VEGF receptor 2 in HUVECs by VEGF was inhibited by **1**, which resulted in the suppression of VEGF-induced activation of downstream signaling molecules, Akt, extracellular signal-regulated kinase 1/2, c-Jun N-terminal kinase, p38, nuclear factor-κB, and matrix metalloproteinase -2 and -9. Elaiophylin down-regulated the expression of VEGF by inhibiting hypoxia inducible factor-1α accumulation in tumor cells. The results demonstrate that **1** blocks tumor cell-induced angiogenesis and may be utilized as a new type of antiangiogenic agent for cancer therapy [40].

### 3.3. Immunosuppressive and Anti-Inflammatory Activity

Elaiophylin (**1**) and 11-O-methylelaiophylin **(2)** showed a potent inhibitory effect (97.5% and 97.6%, respectively) on the activation of B cells by lipopolysaccharide as well as the proliferation of mouse splenic lymphocytes stimulated by mitogens at 1 μg/mL, but 11,11′-O-dimethylelaiophylin (**3**) did not. This result indicated that **1** and **2** would be effective immunosuppressants and 11-OH /11′-OH might be important in immunosuppressive activity [36].

Elaiophylin (**1**), efomycin A (**8**) and G (**9**) were observed to dose-dependently reduce the adhesion of human polymorphonuclear granulocytes to the endothelium. The inhibition rates of adhesion were 93%, 60%, and 95% by **1**, **8**, and **9**, respectively, in a concentration of 5 μM. The results indicated the potential use of **1**, **8**, and **9** for the treatment of acute and chronic inflammations, especially to the use as medicaments in the therapy of myocardial infarct [16].

Efomycins were demonstrated as a new class of selective small-molecule inhibitors of selectin functions. Efomycin M (**17**), which was nontoxic and showed selective inhibitory effects on selectin-mediated leukocyte endothelial adhesion in vitro, significantly diminished rolling in mouse ear venules in vivo. In addition, **17** alleviated cutaneous inflammation in mouse models of psoriasis, one of the most common chronic inflammatory skin disorders. Molecular modeling demonstrated a spatial conformation of **17**, mimicking naturally occurring selectin ligands. Efomycin M might be efficacious in the treatment of human inflammatory disorders [29,41]. Efomycin M could diminish thrombus formation and alleviated myocardial infarction and reperfusion injury by the inhibition of P-selectin–dependent platelet functions [42]. In addition, **17** therapy diminished L-selectin-mediated lymphocyte rolling and T cell mediated-allergic reactions [43]. Efomycin M is currently in preclinical study for the treatment of psoriasis, ischemia-reperfusion, and allergy [44].

### 3.4. Antiviral Activity and α-glucosidase Inhibitory Activity

Lee et al. discovered the antiviral effect of 11-O-methylelaiophylin (**2**) while screening for α-glucosidase inhibition. Compound **2** was found to be an α-glucosidase inhibitor with an IC_50_ value of 10 μM. It showed the mixed type inhibition of α-glucosidase with a Ki value of 5.94 μM. In addition, **2** inhibited the intracellular trafficking of hemagglutinin neuramidase (HN), a glycoprotein of Newcastle disease virus (NDV) in baby hamster kidney (BHK) cells. 11-O-methylelaiophylin inhibited the cell surface expression of NDV-HN glycoprotein without significantly affecting HN glycoprotein synthesis in NDV-infected BHK cells [45]. The connection of antiviral and α-glucosidase inhibitory activity might be explained for the reason that glucosidase inhibitions in the cell bring about an alteration on cell to cell signaling and virus recognition to the cell.

### 3.5. Other Activity

Elaiophylin (**1**), efomycin (**9**), and SNA-4606-1 (**12**) have shown inhibitory activity against testosterone 5a-reductase derived from rat prostate with IC_50_ values of 5.8, 8.7, and 6.6 μM, respectively [25]. Elaiophylin has been found to exert more inhibitory effect than dexamethasone on the synthesis of nitric oxide at non-toxic concentration to macrophage Raw 264.7 cells stimulated with lipopolysaccharide. This indicated that **1** had considerable potential as an inhibitor of NO production, which might have good correlation with the cytotoxic effect and anti- inflammatory activity [33].

## 4. Biosynthesis

The feeding experiments with [1-^13^C, ^18^O_2_]-labeled precursors have revealed that all carbon and oxygen atoms of macrolactone core of elaiophylin (**1**) were derived from PKS precursors [46]. Elaiophylin is assembled by a type I polyketide synthases following the PKS pattern collinearity [1]. Generally, the individual domains in PKSs are grouped into functional modules containing basic modules, such as an acyl-carrier protein transferase domain (AT), an acyl-carrier protein domain (ACP), and a ketosynthase domain (KS), and modification modules, such as a β-ketoreductase domain (KR), a dehydratase domain (DH), and an enoyl reductase domain (ER). The growing polyketide chain transfers from the ACP of the first extender module to the KS of the next module. Once the carbon chain reaches the final extension, the linear polyketide is released by a thioesterase (TE).

**Figure 3:**
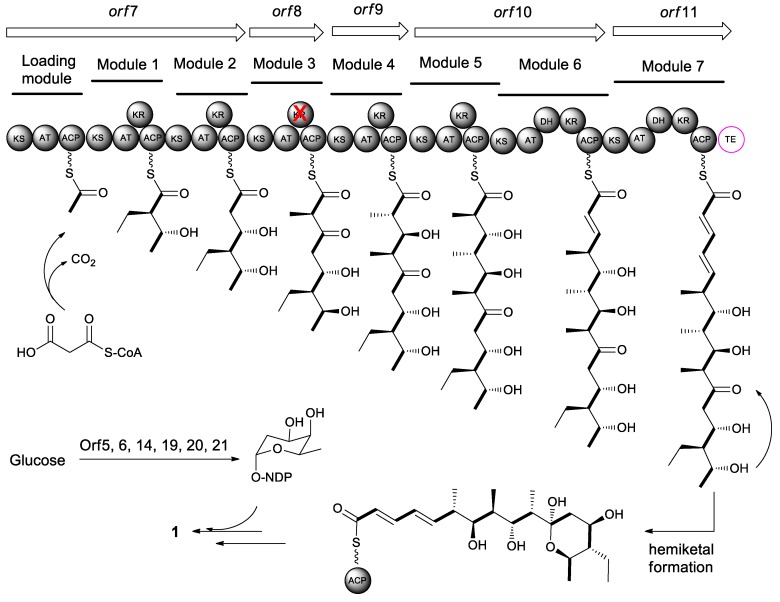
Proposed elaiophylin PKS assembly line.

The elaiophylin biosynthetic gene clusters (*ela*) have been identified in *Streptomyces* sp. DSM4137 [47], *Streptomyces* sp. NRRL 30748 [48], *Streptomyces autolyticus* CGMCC 0516 [49], and *Streptomyces* sp. M56. [31] The *ela* cluster in *Streptomyces* sp. NRRL 30748 comprises 24 individual open reading frames (orf) spanning approximately 63.2 kb DNA. The core cluster consists of five PKSs genes (orf 7–11) that include eight PKS modules in total as well as a free-standing TE domain (Figure 3). Orfs 5, 6, 14, and 19–21 are involved in the biosynthesis and loading of 6-deoxyfucose sugar unit [48]. The alignment of conserved motifs of active site of AT domain in the polyketide backbones is consistent and collinear with the elaiophylin structure [21]. The structural variations, such as the pendant methyl group at C-2/C-2′ and the methyl or ethyl groups at C-14/C-14′ in some elaiophylin analogues, might be due to relaxed substrate specificity of the PKSs that are responsible for elaiophylin biosynthesis in the individual producing strains. A TetR family regulator GdmRIII was found to affect the biosynthetic pathways of both geldanamycin and elaiophylin in the strain of *Streptomyces autolyticus* CGMCC0516. The GdmRIII plays a positive regulatory role in the biosynthesis of geldanamycin, but a negative role in elaiophylin [50].

**Figure 4:**
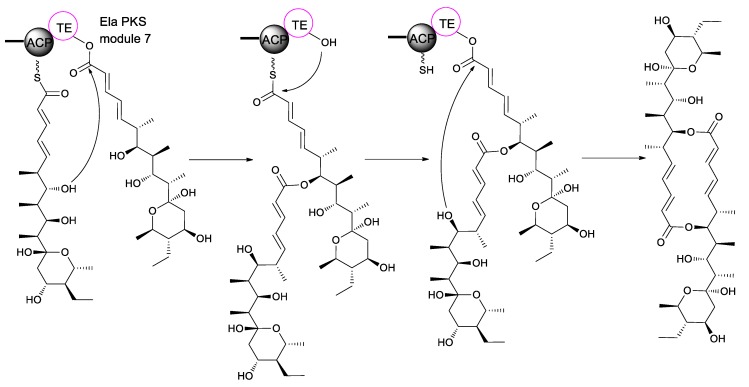
Biosynthetic mechanisms for the formation of elaiophylin diolide.

Biosynthetically, elaiophylin is quite interesting, as it is formed from two linear polyketide precursors, which, when dimerized, produce a cyclic polyketide that possesses a unique C_2_-symmetry that is only found in a small group of polyketides. To probe the mechanism and selectivity of diolide formation, Zhou et al. had constructed ring formation in vitro by using a non-natural substrate [51]. The incubation of recombinant elaiophylin thioesterase with a synthetic analogue of the presumed monomeric polyketide precursor of elaiophylin, specifically its N-acetylcysteamine thioester, produced a novel 16-membered C_2_-symmetric macrodiolide. A linear dimeric thioester is an intermediate in ring formation, which indicates the iterative use of the thioesterase active site in ligation and subsequent cyclization. The iterative *ela*-TE must catalyze a total of two acylation and two deacylation reactions to form the diolide (Figure 4). Furthermore, the *ela* thioesterase acts on a mixture of pentaketide and tetraketide thioesters to give both the symmetric decaketide diolides and the asymmetric hybrid nonaketide diolides. Such thioesterases have potential as tools for in vitro construction of novel diolides.

## 5. Conclusions

This review has highlighted various aspects of elaiophylins, including natural discovery, biological activities, relationship between structure and activity, and biosynthesis. Up to date, 19 natural elaiophylin derivatives have been discovered from different *Streptomyces* spp. Elaiophylins have displayed a wide range of biological properties, especially the remarkable antimicrobial, anticancer, and immunosuppressive activities. Elaiophylins have potent antimicrobial activities against Gram-positive bacteria, even the drug-resistant pathogens, including MRSA and VRE strains, which indicated that elaiophylins might not be affected by the common macrolide resistance mechanism of bacteria. The anticancer effect of elaiophylin (**1**) has been extensively studied that autophagy, inducing apoptosis and proliferation, and antiangiogenic activity were all probably involved. Structure-activity relationship results highlight the importance of the C_2_-symmetric diolide skeleton and the hemiketal moiety with respect to both antibacterial and anticancer activities. As for the aspect of immunosuppressive activity, efomycin M (**17**), without glycosylated hemiketal moiety and with unsaturated enone, is a promising immunosuppressant lead for the treatment of psoriasis, ischemia-reperfusion, and allergy. The *ela* thioesterase is unique in the TE family of polyketide. It has shown great potential as tools to the in vitro construction of novel diolides by acting on a mixture of various pentaketide or tetraketide thioesters. It is also promising to discover new elaiophylin analogues by metabolic engineering or genomic mining with the genomic information of *ela* thioesterase. Hopefully, this review will provide reference for further research interests of these compounds and lay the foundation for new drug discovery.

## Figures and Tables

**Table 1:** Antimicrobial bioassay results (MIC, μg/mL) for **1**–**3**, **9**, **10**, and **11** ^a^.

Strains	Phenotype	1	2	3	9	10	11	Azithromycin	Erythromycin	Oxacillin	Vancomycin
*S. aureus* ATCC 29213	MSSA	1	2	16	2	32	2	2	0.25	0.5	0.5
*S. aureus* 09-6	MSSA	1	2	16	2	32	2	2	0.25	0.5	0.5
*S. aureus* ATCC 33591	MRSA	1	2	16	2	32	2	>256	>256	>256	0.5
*S. aureus* 09-13	MRSA	1	2	16	2	32	2	>256	>256	>256	0.5
*S. aureus* R6101	MRSA	2	1	4	2	16	>64	>256	NT	>256	NT
*S. aureus* ATCC 6538P	Thios-R	0.5	2	16	2	>128	4	>256	NT	NT	NT
*Staphylococcus epidermidis* ATCC 12228	MSSE	1	2	16	4	64	>64	>256	16	>256	0.5
*S. epidermidis* 09-9	MSSE	2	2	16	NT	NT	NT	NT	NT	NT	0.5
*S. epidermidis* 09-3	MRSE	2	2	16	4	64	>64	>256	>256	>256	4
*Enterococcus faecalis* ATCC 29212	VSE	1	32	16	2	64	2	8	16	4	1
*E. faecalis* 09-8	VSE	1	2	16	16	64	2	>256	32	>256	1
*E. faecalis* ATCC 51299	VRE	1	1	16	2	64	2	>256	8	>256	4
*E. faecalis* W4138	VRE	1	2	16	4	64	4	>256	NT	NT	4
*E. faecalis* R6512	VRE	1	1	8	2	>128	4	>256	NT	NT	>256
*Enterococcus faecium* 09-10	VSE	2	2	32	>64	64	>64	>256	>256	>256	1
*E. faecium* ATCC 700221	VRE	1	2	16	2	64	4	>256	64	>256	128
*Micrococcus faecium* ATCC 10240	Apram-R	0.5	2	16	2	128	2	2	NT	NT	NT
*Escherichia coli* 09-1	ESBL-prod	>256	>256	>256	>64	>128	>64	64	>256	>256	>256
*E. coli* 09-20	BL-prod	>256	>256	>256	>64	>128	>64	>256	>256	>256	>128
*Klebsiella pneumoniae* ATCC 700603	ESBL-prod	>256	>256	>256	>64	>128	>64	64	>256	>256	>128
*K. pneumoniae* ATCC BAA-2146	NDM-1-prod	>256	>256	>256	>64	>128	>64	64	>256	>256	>128
*Pseudomonas aeruginosa* ATCC 27853	BL-prod	>256	>256	>256	>64	>128	>64	256	>256	>256	>128
*Morganella morganii* ATCC 25830	BL-prod	>256	>256	>256	>64	>128	>64	64	>256	256	128

^a^ Abbreviations: Methicillin-susceptible *S. aureus*. (MSSA), Methicillin-resistant *S. aureus* (MRSA). Not tested (NT). Thiostrepton-resistant (Thios-R). Methicillin-susceptible *S. epidermidis* (MSSE). Methicillin-resistant *S. epidermidis* (MRSE). Vancomycin-susceptible *Enterococcus* (VSE). Vancomycin-resistant *Enterococcus* (MRE). Apramycin-resistant (Apram-R). Extended spectrum *β*-lactamase (ESBL). *β*-lactamase (BL). New Delhi metallo-*β*-lactamase 1 (NDM-1).
